# Crosstalk Between Metabolic Dysfunction-Associated Steatotic Liver Disease and Atrial Fibrillation: Shared Mechanism, Diagnostic Integration, and Management Implications

**DOI:** 10.3390/life15111713

**Published:** 2025-11-05

**Authors:** Agata Morawska, Rafał Frankowski, Mikołaj Grabarczyk, Marcin Kosmalski, Monika Różycka-Kosmalska

**Affiliations:** 1 Students’ Research Club, Department of Clinical Pharmacology, Medical University of Lodz, 90-153 Lodz, Poland; agata.morawska@student.umed.lodz.pl; 2 Department of Clinical Pharmacology, Medical University of Lodz, 90-153 Lodz, Poland; rafal.frankowski@student.umed.lodz.pl (R.F.); mikolaj.grabarczyk123@wp.pl (M.G.); marcin.kosmalski@umed.lodz.pl (M.K.); 3 Department of Electrocardiology, Medical University of Lodz, 92-213 Lodz, Poland

**Keywords:** metabolic-associated fatty liver disease, atrial fibrillation, pathogenesis, diagnosis, treatment

## Abstract

Metabolic dysfunction-associated steatotic liver disease (MASLD) and atrial fibrillation (AF) are two highly prevalent conditions that share overlapping cardiometabolic risk factors, including obesity, type 2 diabetes, hypertension, and dyslipidemia. Growing evidence suggests that these two disease entities are pathophysiologically linked through systemic inflammation, oxidative stress, and structural remodeling. Population-based studies and meta-analyses report an association between steatotic liver disease and both incident and recurrent AF. While several analyses observe persistence of this association after adjustment for cardiometabolic risk factors, residual confounding and limitations of observational designs preclude firm causal inference. Conversely, heart rhythm disturbances may exacerbate hepatic fibrosis and dysfunction. Lifestyle interventions—particularly sustained weight loss—have demonstrated significant benefits in both conditions. Emerging pharmacological options, including incretin mimetics, flozins, statins, and thiazolidinediones, show promise in addressing the liver–heart axis, while appropriate anticoagulation remains essential in AF management. This review summarizes current epidemiological data, mechanistic insights, diagnostic approaches, and therapeutic strategies related to the coexistence of MASLD and AF. Emphasis is placed on shared pathogenic pathways, non-invasive diagnostic tools, and integrated management options.

## 1. Introduction

Metabolic dysfunction-associated steatotic liver disease (MASLD) is a chronic liver disease associated with metabolic syndrome and represents a threat to global and public health due to its complications. MASLD is the most common liver impairment in the general population and, furthermore, the leading cause of mortality and morbidity related to liver diseases [1]. MASLD is a fatty liver condition characterized by the presence of one or more cardiometabolic risk factors but without harmful effects exerted by alcohol intake; consequently, these individuals face a higher risk of cardiovascular disease (CVD) [2]. Recently, MASLD has replaced the term non-alcoholic fatty liver disease (NAFLD), which was used as previous name of this pathological condition [3]. To maintain consistent terminology, this review utilizes the MASLD term, with exception for studies and trials based on legacy cohorts, where the NAFLD term was retained. The prevalence of MASLD is steadily increasing, with an estimated 30% of the world’s adult population currently affected [1]. Moreover, around 75% of individuals with excess body weight develop MASLD, linking its prevalence to global obesity trends [4]. The pathogenesis of MASLD is complex. Among the risk factors of this condition are dyslipidemia, obesity, hypertension (HA), insulin resistance (IR), and hypertriglyceridemia [5,6]. The development of MASLD and its progression to metabolic-associated steatohepatitis (MASH) can be explained by the interplay of lipid metabolism pathways [7]. Its complications encompass MASH, cirrhosis, and hepatocellular carcinoma [8,9]. Moreover, MASLD increases cardiovascular risk and the associated consequences [10]. Indeed, CVD incidence is elevated among MASLD patients, and epidemiological statistics acknowledge it as the leading cause of mortality in this group [11,12]. Conversely, some studies have suggested that MASLD per se does not increase mortality risk in individuals with cardiometabolic risk factors, except among those with higher alcohol consumption [13].

Atrial fibrillation (AF) is a highly prevalent age-related cardiac arrhythmia, and its occurrence is associated with increased morbidity and mortality [14]. According to the 2024 ESC Guidelines, AF is one of the most common heart rhythm disorders. Mechanistically, this disease is characterized by disorganized electrical impulses in the atria, which result in ineffective and rapid atrial contractions [15,16]. As a supraventricular arrhythmia with uncoordinated atrial activation, AF results in a loss of effective atrial contraction. The distinctive features of surface electrocardiogram (ECG) in this condition include the absence of discernible and regular P waves as well as irregular activation of the ventricles. This results in no specific pattern to RR intervals in the absence of an atrioventricular block [15,16]. The irregular rhythm generated in AF leads to disrupted and turbulent blood flow within the heart, creating favorable conditions for the formation of thrombi (blood clots). These clots may subsequently embolize and result in a stroke. AF is recognized as the primary cardiac-related cause of ischemic stroke. AF is also a major risk factor for other forms of CVD, significantly elevating the odds of heart failure (HF), sudden cardiac death, and overall cardiovascular mortality [17]. Current practice guidelines identify modifiable risk factors for AF as obesity, HA, sedentary lifestyle, sleep apnea, smoking, IR, and type 2 diabetes mellitus (T2DM). As referenced above, similar conditions also contribute to the development of MASLD. Moreover, AF is characterized by disorganized atrial electrical activity, which leads to persistent hemodynamic fluctuations and structural remodeling of the heart; these processes may be exacerbated by metabolic dysfunction, such as MASLD [17,18].

Given the increasing burden of MASLD and the shared risk factors with AF, this study aims to characterize the coexistence of MASLD and AF from a clinical perspective, focusing on epidemiological patterns, diagnostic pathways, and multidisciplinary treatment options, considering the benefits and risks of drug use. The overarching aim is to translate these insights into actionable clinical recommendations.

## 2. Epidemiological Data on Coexistence of MASLD and AF

### 2.1. Risk of AF Development in MASLD-Affected Individuals

MASLD is currently estimated to affect approximately 38% of the global population [19,20]. Worldwide MASLD prevalence has risen by over 50% in the last three decades, increasing from 25.3% (1990–2006) to 38.0% (2016–2019) according to a recent systematic review, highlighting MASLD’s expanding public health burden and the need for proactive clinical and research responses [21]. The global rise in MASLD occurrence mirrors trends in obesity and T2DM, underscoring their shared metabolic origins [19,22].

The global prevalence of AF has risen significantly over the past 30 years, now affecting an estimated 60 million people worldwide. The lifetime risk of AF is around 33%, with individual estimates influenced by factors such as age, sex, race, and the presence of clinical risk factors [23]. In a prospective multicenter cohort study including patients with nonvalvular AF, NAFLD was diagnosed in 732 of 1735 (42.2%) participants. Patients with NAFLD were younger, less frequently women, and more likely to be treated with non-vitamin K oral anticoagulants (NOACs) or to have obesity, dyslipidemia, and persistent/permanent AF [24]. In a similar analysis, subjects diagnosed with MASLD were more likely than controls to present with T2DM, obesity, HA, dyslipidemia, and chronic kidney disease (CKD). Coexistence of T2DM markedly amplified the risk of cardiovascular events, malignancy, and liver-related complications in individuals with MASLD [25]. Moreover, they exhibited a modest but statistically significant increase in the incidence of AF compared with controls (difference: 0.9 per 1000 person-years) [26]. Another assessment revealed that among patients with biopsy-confirmed non-alcoholic steatohepatitis (NASH), the prevalence of AF was roughly double that of the general population [27]. Screening of 6293 individuals, suffering from NAFLD, allowed the detection AF in 59 patients (0.9%). Compared with those without AF, affected subjects were significantly older (64.6 vs. 52.0 years, *p* < 0.001), had a higher body mass index (BMI) (26.6 vs. 25.2 kg/m^2^, *p* < 0.001), and a larger waist circumference (89.9 vs. 84.0 cm, *p* < 0.001). Notably, AF was independently associated with advanced liver fibrosis in this cohort. Using the NAFLD fibrosis score (NFS), AF remained a significant predictor in both the low cut-off value (COV) group (adjusted odds ratio (aOR) = 2.85, *p* = 0.004) and high-COV group (aOR = 12.29, *p* < 0.001). Similarly, when assessed by the fibrosis-4 (FIB-4) index, AF was independently associated with advanced fibrosis in both the low-COV (aOR = 2.49, *p* < 0.001) and high-COV groups (aOR = 3.84, *p* = 0.016). These findings highlight a significant correlation between AF and fibrosis severity in patients with MASLD [17]. Kim et al. in a nationwide population-based study showed that over a median follow-up of 9.6 years, AF was newly diagnosed in 5335 participants, corresponding to an incidence rate of 2.74 per 1000 person-years. The risk of AF was significantly elevated among patients with MASLD who abstained from alcohol (adjusted hazard ratio (aHR) 1.32, *p* < 0.001), as well as in those with MASLD and concomitant alcohol consumption (aHR 1.48, *p* < 0.001). Notably, when compared with all other alcohol consumers regardless of steatotic liver disease status, nondrinking patients with MASLD demonstrated a persistently higher risk of AF (aHR 1.11, *p* = 0.011) [27]. Cho et al. presented a nationwide study, in which participants with MASLD exhibited an elevated risk of new-onset AF (aHR 1.10). Those with MASLD combined with additional metabolic or clinical comorbidities demonstrated an even higher risk of AF development (aHR 1.22) [28]. Consistent findings come from meta-analyses of large cohort studies. Evaluation of nine cross-sectional and longitudinal studies, including over 360,000 subjects revealed a strong correlation: NAFLD doubles the odds of prevalent AF (OR ~2.07) [29]. An even larger examination, comprising data gathered from 18 million participants, reported a risk ratio (RR) of 1.42 for AF, HF, stroke, and cardiovascular mortality among NAFLD patients [30]. A meta-analysis conducted by Mantovani et al. in 2024, after identifying 16 retrospective cohort studies with aggregate data on ~19.5 million individuals followed for a median of 7.2 years, showed that MASLD was significantly associated with increased odds of developing incident AF (random-effects hazard ratio (HRRE) 1.20). This correlation did not appear to increase further with the severity of liver fibrosis. The risk of AF remained significant even after adjusting for age, sex, BMI, HA, T2DM, or other cardiometabolic risk factors. Sensitivity analyses did not modify these findings. However, the collective data regarding this particular association come from observational studies, with important limitations resulting from their design as well as heterogeneity of examined populations. Therefore, the observations that MASLD exacerbates AF risk independently from fundamental cardiometabolic factors shall be taken with caution [31]. Moreover, in one study, the presence of MASLD also significantly increased the risk of AF recurrence after cryoballoon ablation, underscoring the importance of targeted interventions for MASLD in the periprocedural management of AF [32].

### 2.2. Controversies and Evidence Appraisal

Across cohorts, effect estimates vary by disease definitions (NAFLD vs. MASLD), exposure ascertainment (enzymes vs. imaging vs. histology), and AF endpoints (incident vs. prevalent vs. post-ablation recurrence). Associations typically attenuate with broader adjustment for adiposity and diabetes, and residual confounding and reverse causality remain possible. Prior meta-analyses differ in inclusion criteria and adjustment sets; a side-by-side summary is provided in Table 1. Collectively, current data support an association but not definitive independence or causality.

Across meta-analyses [30,31], MASLD/NAFLD is associated with a modestly increased risk of incident AF (11–20% after full adjustment), converging with biopsy-based evidence [25,26]. Estimates diverge in international classification of diseases (ICD)-only cohorts or when AF is not a primary endpoint. Adjustments for adiposity, T2DM, and HA attenuate, but do not abolish, the association; fibrosis severity yields higher risks in some cohorts (e.g., cirrhosis), while ablation populations show stronger effects for recurrence [32].

## 3. Pathophysiological Mechanisms Contributing to Development and Progression of MASLD and AF

**Figure 1:**
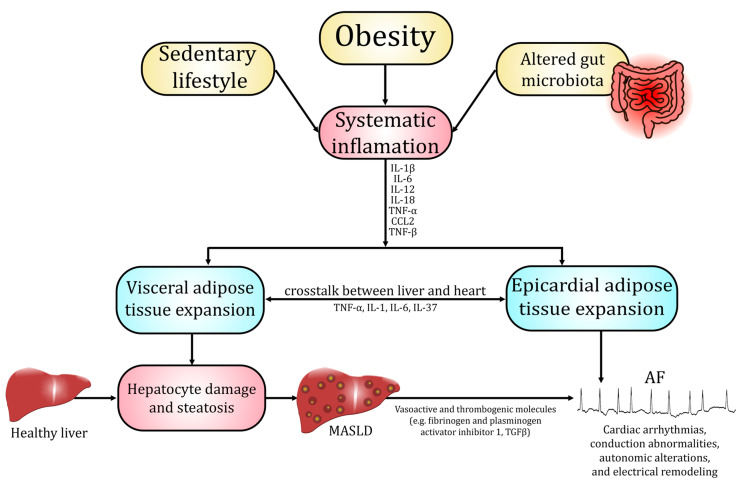
Pathophysiological and biochemical mechanisms linking MASLD and AF.

Several population-based cohort studies, which utilized mildly elevated serum liver enzyme levels as surrogate indicators of MASLD, a method that warrants cautious interpretation, demonstrated that this disorder was independently associated with an increased long-term risk of developing AF [34]. Moreover, advanced MASLD significantly contributed to occurrence of AF compared with no-MASLD [35]. Concurrent MASLD and AF independently and additively elevate cardiovascular risk. Moreover, AF in MASLD patients is linked to an increased incidence of HF, stroke, and CVD-associated death [36]. CVD, including AF and HF, is the leading cause of death in patients with MASLD, followed sequentially by extrahepatic malignancies and complications directly related to hepatic dysfunction [11,13,37,38,39]. This section summarizes data regarding pathophysiological pathways and molecular alterations that promote development and progression of MASLD and AF. Most essential pathophysiological and biochemical mechanisms linking development of MASLD and AF have been summarized in Figure 1.

### 3.1. Inflammatory and Oxidative-Stress Pathways

The development of MASLD is believed to be influenced by a range of interconnected physiological disturbances, with chronic systemic inflammation and oxidative stress as key factors. Excessive lipid accumulation in hepatocytes enhances mitochondrial β-oxidation and cytochrome P450 activity, leading to the overproduction of ROS, which overwhelm antioxidant defense systems such as superoxide dismutase (SOD), catalase (CAT), and glutathione peroxidase (GPX) [6,40]. The ensuing oxidative imbalance further induces lipid peroxidation, protein oxidation, and mitochondrial dysfunction, activating stress-responsive pathways including nuclear factor kappa β (NF-κβ) and c-Jun N-terminal kinase (JNK) that promote the transcription of tumor necrosis factor α (TNF-α), interleukin (IL)-6, and IL-1β [41,42]. These cytokines amplify hepatic inflammation by recruiting immune cells and stimulating Kupffer cell activation, while simultaneously triggering hepatic stellate cell activation and fibrogenesis [41]. Antioxidant dysregulation—particularly impairment of the nuclear factor erythroid 2-related factor 2 (Nrf2)–GPX4 axis—further exacerbates oxidative injury and lipid peroxidation, contributing to hepatocellular damage and disease progression [6,40]. Persistent oxidative and inflammatory signaling thus drives the transition from simple steatosis to MASH, ultimately predisposing to cirrhosis and hepatocellular carcinoma [6,41,43]. Oxidative stress and chronic, low-grade inflammation in MASLD not only drive hepatic injury but also generate systemic mediators that promote atrial structural and electrical remodeling conducive to AF [44,45]. Circulating oxidative byproducts and impaired systemic antioxidant capacity exert unfavorable alterations in the myocardium and provoke atrial myocyte oxidative damage, mitochondrial dysfunction, and abnormal Ca^2+^ handling—all established triggers of AF [44,45]. Concurrently cytokines such as IL-1β, IL-6, and TNF-α induce endocrine and paracrine effects on the heart, which result in atrial fibroblast activation and extracellular matrix deposition. Induction of the NLR family pyrin domain containing 3 (NLRP3)/caspase-1 axis also contributes directly to cardiomyocyte electrical instability by altering sarcoplasmic reticulum Ca^2+^ release and promoting spontaneous discharges [46,47,48].

### 3.2. Adipose Tissue and Epicardial Fat Signaling

Dysfunctional white adipose tissue acts as an endocrine organ that drives MASLD progression through several coordinated alterations. Hypertrophic adipocytes and insulin resistance increase lipolysis and the release of free fatty acids to the liver. Simultaneously, an altered secretome (decreased hepatoprotective adiponectin and omentin and increased pro-inflammatory adipokines such as leptin, resistin, chemerin, visfatin, and elevated chemokines (mostly monocyte chemoattractant protein 1 (MCP-1))) promotes hepatic steatosis, further impairs sensitivity to insulin, as well as induces Kupffer cell activation and fibrogenesis [49,50,51,52]. Chronic adipose inflammation with macrophage infiltration and increased lipolytic flux not only supplies hepatotoxic lipid species (diacylglycerols, ceramides) but also reduces systemic antioxidant capacity and raises circulating cytokines (IL-6, TNF-α, IL-1β) that exacerbate dysfunction of hepatic tissue [53,54]. These adipose-derived pathways extend to the heart through visceral and ectopic depots, most notably epicardial adipose tissue (EAT), which—by virtue of its anatomic contiguity and paracrine secretory activity—conveys adipokines, cytokines, free fatty acids, and extracellular vesicles directly to the atrial myocardium [55,56]. In obese and metabolically dysfunctional states, EAT becomes inflamed and profibrotic (higher production of local leptin, chemerin, IL-6, IL-1β, transforming growth factor β (TGF-β), and matrix-remodeling proteases), driving atrial fibroblast activation, extracellular-matrix (ECM) deposition, and fibrosis [55,57,58]. It also perturbs cardiomyocyte electrophysiology through oxidative stress, altered Ca^2+^ handling, and ion-channel modulation, thereby increasing atrial ectopy and promoting the development of AF [55,56,57]. Clinical and imaging studies show that increased EAT volume and altered EAT phenotype correlate with MASLD severity and with higher incidence and recurrence of AF after ablation, supporting a liver/adipose tissue/heart axis in which adipose endocrine dysfunction links MASLD pathobiology to arrhythmogenesis [50,56,58].

### 3.3. Fibrosis and Structural Remodeling (Atrial Cardiomiopathy)

Progression of MASLD to advanced fibrosis and cirrhosis is driven by a sustained wound-healing response in which chronic hepatocellular injury, inflammation, and metabolic stress activate hepatic stellate cells and portal fibroblasts to produce excess ECM components, principally fibrillar collagens (type I and III), fibronectin, and proteoglycans, while the balance between matrix metalloproteinases (MMPs) and tissue inhibitors of metalloproteinases inhibitors (TIMPs) shifts toward decreased matrix degradation, causing ECM accumulation and increased tissue stiffness [59,60,61]. This profibrotic environment is orchestrated by paracrine mediators (notably TGF-β and platelet-derived growth factor (PDGF)), macrophage-derived signals, and hepatocyte senescence/damage-associated molecular pattern (DAMP) signaling that sustain hepatic stellate cell activation and myofibroblast transdifferentiation [59,60]. Concurrently, altered ECM composition and crosslinking, including the upregulation of lipoxygenase (LOX) family enzymes and galectin-3–associated pathways, create a mechanically and biochemically abnormal niche that reinforces fibrogenesis and impairs liver function [59,60,62]. The same systemic and paracrine profibrotic mechanisms that remodel the liver can promote structural remodeling of the heart and contribute to atrial cardiomyopathy [63,64]. Chronic hepatic fibrosis is associated with persistent systemic inflammation and elevated circulating profibrotic mediators (TGF-β, galectin-3) and ECM fragments that can reach the myocardium, where they promote fibroblast activation and shift cardiac MMP/TIMP balance toward ECM deposition. In the atria, this leads to interstitial fibrosis, conduction heterogeneity, and impaired contractile function—hallmarks of atrial cardiomyopathy [44,60,62,63,64]. These alterations combined strongly contribute to development and aggravation of AF. Recent mechanistic and clinical studies link higher liver fibrosis burden or fibrosis biomarkers with greater prevalence and incidence of AF as well as with poorer outcomes of this disease entity [63,64,65]. This conceptual integration reframes AF not merely as an electrical disorder but as a manifestation of multisystem fibrotic remodeling consistent with the atrial cardiomyopathy paradigm [63,64].

### 3.4. Genetic and Molecular Mediators

Genetic variation and molecular regulatory mechanisms are major determinants of the susceptibility to and progression of MASLD. Common single-nucleotide polymorphisms in genes controlling hepatic lipid and retinol metabolism, notably *PNPLA3*, *TM6SF2*, *GCKR,* and *MBOAT7*, increase fat retention in liver, promote lipotoxicity, and accelerate inflammation-driven fibrosis. Loss-of-function variants in *HSD17B13* are protective against progression to steatohepatitis and fibrosis [66,67,68]. Large genome-wide association studies (GWASs) and multi-omics studies further show that these loci influence hepatic gene expression, lipid and retinoid pathways, as well as mitochondrial/metabolic networks that determine whether simple steatosis progresses to inflammatory, fibrotic disease [66,68]. Beyond inherited variation, epigenetic remodeling (DNA methylation, histone modifications) and noncoding RNAs—especially extracellular-vesicle–packaged miRNAs that regulate inflammation, insulin signaling, and fibrogenic programs—act as dynamic molecular mediators linking environmental exposures (diet, obesity) to MASLD phenotypes [69,70]. These genetic and molecular mechanisms plausibly increase the risk of AF through shared downstream biology rather than by simple gene-for-gene causation. The *PNPLA3*/*TM6SF2*-driven propensity to hepatic lipid accumulation, mitochondrial dysfunction, and inflammation promotes systemic metabolic derangement, chronic low-grade inflammation, and profibrotic circulating mediators, including TGF-β and ECM fragments, which can reach the myocardium and foster atrial fibrosis and electrical remodeling—core components of atrial cardiomyopathy [64]. Observational cohorts show that MASLD severity predicts a higher incidence of new-onset AF [44]. By contrast, GWAS of AF have identified largely distinct cardiac loci, primarily involving genes related to ion-channel regulation (*PITX2*, *KCNN3*), cardiac structural proteins (*TTN*, *MYH6*), and transcriptional networks of atrial development [71]. Importantly, variants referenced above, strongly associated with hepatic lipid metabolism and MASLD susceptibility, do not significantly overlap with these AF risk loci, indicating that the genetic architecture of AF is primarily cardiac, whereas MASLD increases AF risk through systemic metabolic and inflammatory pathways rather than direct shared heritability [71,72].

## 4. Diagnostic Measures for Detection of MASLD and AF

The gold standard for diagnosing MASLD remains liver biopsy, which enables detection of increased fat accumulation within hepatic tissue. Results of histopathological assessment combined with the presence of at least one of five cardiometabolic risk factors—obesity, hyperglycemia, HA, hypertriglyceridemia, or hypoalphalipoproteinemia—and ruling out excessive consumption of alcohol and other chronic liver diseases allow the fulfillment of the diagnostic criteria and confirmation of the presence of this metabolic disorder [73]. However, due to biopsy’s invasive nature and associated discomfort, it is often not well tolerated by patients. As a result, non-invasive imaging techniques such as magnetic resonance imaging–proton density fat fraction (MRI-PDFF) and transient elastography (TE) have become widely utilized for the detection of hepatic steatosis and steatohepatitis. Among these, TE is gaining prominence owing to its high sensitivity and specificity in identifying MASLD, coupled with a high rate of patient acceptance. Diagnosis based on combination of biopsy and radiological imaging is limited in availability and costly [74]. To address this challenge, researchers have introduced a simple, non-invasive testing (NIT) approach that relies on biochemical blood tests and scoring systems [65]. FIB-4 index, calculated from aspartate aminotransferase (AST), alanine aminotransferase (ALT), platelet count, and patient age has emerged as a practical first-line tool in primary care for identifying advanced fibrosis in individuals with MASLD. Its simplicity, low cost, and ability to stratify risk make it a gateway test that can determine which patients require more specialized evaluation [75]. Another relatively simple and therefore widely used blood-based test is NFS. It allows the estimation of the likelihood of advanced fibrosis using parameters like BMI, fasting blood glucose, and liver enzymes. Recently AST/platelet ratio index (APRI), initially employed for detecting advanced fibrosis in patients with chronic hepatitis C virus, has also become more commonly used among individuals with MASLD. However, it should be noted that its usefulness as a standalone diagnostic measure is limited, and it is considered unsuitable for identifying significant fibrosis alone. A more complex and advanced approach utilizes the enhanced liver fibrosis (ELF) test. This diagnostic tool estimates the severity of liver fibrosis by measuring hyaluronic acid, procollagen III amino-terminal peptide (PIIINP), and TIMP-1—serum markers of changes occurring in the extracellular matrix during the fibrogenesis process. Other blood-derived indicators of fibrosis progression are type IV collagen 7s domain and Mac-2 binding protein glycosylation isomer (M2BPGi), although they are not a part of the ELF test panel [75,76,77]. Apart from scoring systems developed to improve the diagnostic process, there are several additional biomarkers that may serve as accessory factors highlighting deterioration of organism’s metabolic condition and therefore, progression of liver disease. Among the most important ones are the triglyceride–glucose index (TyG), triglyceride–glucose–body mass index (TyG-BMI), atherogenic index of plasma (AIP), non-high-density lipoprotein cholesterol to high-density lipoprotein cholesterol ratio (NHHR), uric acid to albumin ratio (UAR), and uric acid to high-density lipoprotein cholesterol ratio (UHR) [65]. The monocyte-to-high-density lipoprotein cholesterol ratio (MHR), red cell distribution width-to-albumin ratio (RAR), neutrophil-to-lymphocyte Ratio (NLR), systemic inflammatory response index (SIRI), neutrophil-to-platelet ratio (NPAR), and prognostic nutritional index (PNI) are emerging biomarkers that reflect both inflammatory activity and nutritional status [78,79]. As excessive inflammatory activity and impaired carbohydrate or lipid metabolism negatively affect functionality of both liver and heart, many of the abovementioned markers and scoring systems may also be utilized as additional tools for predicting risk of cardiovascular events and disorders [65,80,81]. For instance, individuals with higher values achieved in FIB-4 or NFS indices are simultaneously at greater risk of developing AF and display higher all-cause mortality because of more frequent adverse outcomes like major bleeding or heart failure [80,81]. The fatty liver index (FLI) is the most well-validated tool for ruling in or ruling out hepatic steatosis in older adults and has also been shown to predict the incidence of AF [82]. Considering these findings, it may seem reasonable to screen patients with MASLD or other forms of metabolic disorders for the presence of different kinds of arrhythmias, especially AF.

**Figure 2:**
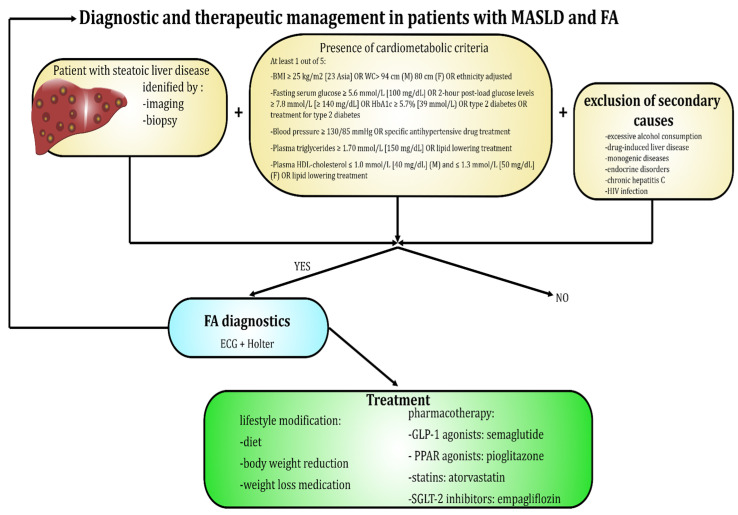
Diagnostic and therapeutic options available in patients suffering from MASLD and AF.

**Figure 3:**
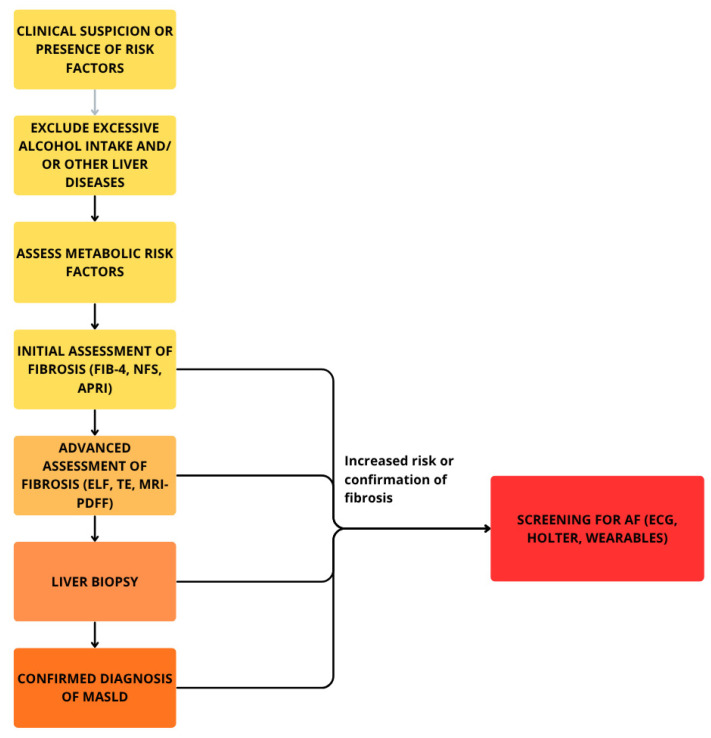
Diagnostic scheme for detection of MASLD and accompanying AF.

In routine clinical practice, the diagnosis of AF does not pose a significant challenge, as it relies on simple and widely accessible methods, such as pulse palpation and ECG-based devices, including the standard 12-lead electrocardiogram and Holter monitoring (ranging from 24 h to a week or longer) [83]. Characteristic features of ECG in AF include lack of discernible and regular P waves and no specific pattern to RR intervals [16]. Figure 2 and Figure 3 present a diagnostic scheme for the detection of MASLD and AF as well as an overview of the most common diagnostic and therapeutic measures available in these disease entities.

## 5. Potential Therapeutic Strategies Targeting MASLD and Atrial Fibrillation

### 5.1. Interventions Affecting Lifestyle and Body Weight

Lifestyle interventions may provide a dual advantage, simultaneously attenuating the progression of MASLD while reducing the risk and burden of AF [84]. Sustained weight loss (~7–10%) improves steatosis, inflammation, and fibrosis [26]. Overall, the findings from Chenug et al. studies demonstrate significant positive associations between MASLD and AF, indicating that managing MASLD, traditionally considered a relatively benign condition, may play a preventive or therapeutic role in AF development. Despite some heterogeneity across studies, clinical trials consistently demonstrate that interventions focusing on weight loss are associated with improvement in hepatic biomarkers among individuals with MASLD in the short to intermediate term. However, data on long-term clinical outcomes remain limited. These findings underscore the importance of revising current clinical guidelines to incorporate structured weight management programs as a core component of MASLD management [85]. Lifestyle and risk factor modification (LRFM) has been increasingly recognized as a complementary pillar of AF management, standing alongside rhythm control, rate control, and anticoagulation [32]. On the subject of the AF, the Long-Term Effect of Goal-Directed Weight Management in an Atrial Fibrillation Cohort (LEGACY) study claims that sustained long-term weight reduction has been consistently linked to a marked decrease in AF burden and an improved likelihood of maintaining durable sinus rhythm [86]. Weight reduction achieved through a low-calorie diet and structured exercise has been shown to lower systemic inflammatory markers, including IL-6 and TNF-α [87]. In a meta-analysis of 116 studies, Askarpour et al. demonstrated that bariatric surgery in obese patients was associated with significant reduction in levels of inflammatory biomarkers, including CRP (*p* < 0.001), IL-6 (*p* < 0.001), and TNF-α (*p* = 0.031) [88]. As systemic inflammatory state is an important condition contributing to development and progression of both MASLD and AF, the interventions focused on its alleviation emerge as effective elements of wide-ranging prophylaxis and treatment in these two disease entities [87,88].

### 5.2. Pharmacotherapeutic Options—Where Are We Today?

Currently, no pharmacological agents have been approved specifically for the treatment of MASLD/NAFLD [82]. Various drugs are being investigated, including incretin mimetics, SGLT-2 inhibitors, pioglitazone, and statins. These agents may also confer benefits in the treatment or prevention of AF.

#### 5.2.1. GLP-1 Agonists

Some of the available treatment options utilize the beneficial potential of incretins and the gut–liver axis [89]. Gut-associated signals encompass a broad spectrum of factors, including dietary nutrients, enteroendocrine hormones, bile acids, microbial metabolites, and antigens. These signals play a pivotal role in modulating immune responses and systemic metabolic processes, underscoring the gut’s function as a key integrative organ, regulating metabolism, and immunity [90]. Incretins, such as glucagon-like peptide-1 (GLP-1) and glucose-dependent insulinotropic polypeptide (GIP), are metabolic hormones released postprandially by specialized enteroendocrine cells within the gastrointestinal mucosa. These hormones play a crucial role in glycemic regulation by enhancing insulin secretion and suppressing glucagon release from pancreatic alpha cells. Additionally, they contribute to delayed gastric emptying and are thought to promote central glucose clearance, potentially leading to reduced appetite and food intake. Pharmacological agents that mimic incretin activity or combine actions of multiple incretins have demonstrated significant metabolic benefits, including improved glycemic control, enhanced insulin sensitivity, reduction in hepatic steatosis, and meaningful weight loss [91]. GLP-1 receptor agonists such as dulaglutide, exenatide, liraglutide, or semaglutide have been licensed for the treatment of T2DM and, more recently, obesity, two major risk factors in AF [92]. GLP-1 receptor agonists, approved for T2DM and obesity, were among the first agents tested in MASLD, with positive phase 2 results leading to phase 3 trials [36]. In the Liraglutide Safety and Efficacy in Patients with Non-alcoholic Steatohepatitis (LEAN) trial (NCT01237119), liraglutide achieved histological resolution of MASH without worsening fibrosis. These effects were accompanied by improvements in metabolic parameters, including enhanced insulin sensitivity and reduced lipotoxicity [93]. Similarly, semaglutide, an approved anti-diabetic agent, has shown promising MASH resolution but limited effects on fibrosis improvement [91]. GLP-1 receptor agonists reduce AF risk by stabilizing glucose metabolism, decreasing epicardial fat, controlling weight, regulating calcium levels, lowering oxidative stress, and modulating blood pressure. Weight loss is mediated via brown adipose tissue thermogenesis and neuropeptide Y (NPY)/agouti-related protein (AgRP) signaling, while oxidative stress is mitigated through connective tissue growth factor (CTGF) suppression and advanced glycation end products (AGE)/receptor of advanced glycation end products (RAGE) inhibition [94]. Meta-analysis of 10 randomized clinical trials demonstrated that semaglutide significantly reduced the incidence of AF by 42% compared with placebo in individuals at high cardiovascular risk, predominantly those with T2DM. This effect was consistent regardless of the drug’s route of administration (oral or subcutaneous), the presence of T2DM, or BMI [95]. Utilization of GLP-1 receptor agonist was also associated with a reduced risk of AF recurrence in patients undergoing AF ablation [96]. Similar results, regarding reduced risk of AF, were also noted in a matched cohort of 14,566 GLP-1 receptor agonists (RAs) and dipeptidyl peptidase 4 (DPP-4) inhibitor pairs, followed for a median of 3.8 years. However, the protective effect was significant only for participants receiving GLP-1RA [97]. Table 2 presents summarized results from studies regarding GLP-1RA effects in MASLD and AF.

#### 5.2.2. PPAR Agonists—Thiazolidinediones

In a randomized, double-blind, placebo-controlled trial investigating the effects of pioglitazone, patients were assigned to receive either pioglitazone or a placebo over a 6-month period. Liver biopsies were conducted both before and after treatment to assess histological changes. The study included 26 patients treated with pioglitazone (mean age 51 ± 7 years; 53.8% men) and 21 patients receiving a placebo (mean age 51 ± 10 years; 33% men). Compared with placebo, the pioglitazone group demonstrated significant improvements in key liver histological features, including steatosis (*p* = 0.003), ballooning necrosis (*p* = 0.02), and inflammation (*p* = 0.008). These findings suggest that pioglitazone may effectively reduce liver injury and inflammation in affected patients [98]. A meta-analysis of four randomized controlled trials, across three continents, demonstrated that pioglitazone significantly improved liver fibrosis in patients with NASH compared w controls (OR 1.7; 95% CI, 1.0–2.8). Moreover, thiazolidinediones, in general, markedly alleviated ballooning degeneration, lobular inflammation, steatosis, and combined necroinflammation in patients with NASH [99]. A meta-analysis by Zhang et al., including three randomized controlled trials (RCTs) and four observational studies with 130,854 patients, found that thiazolidinedione therapy was associated with a 30% lower risk of AF compared with controls (OR 0.73; 95% CI, 0.62–0.87) [100]. In a prospective observational cohort of 150 patients with drug-refractory paroxysmal atrial fibrillation (PAF) and T2DM undergoing catheter ablation, pioglitazone was associated with improved maintenance of sinus rhythm and a lower rate of repeat ablation [101]. Mechanistic explanation for pioglitazone-induced protection against AF comes from an in vivo study, which demonstrated that this drug significantly reversed β1-Aab-induced AF susceptibility, improved atrial structural remodeling, reduced systemic IR, and enhanced the expression of key glycolipid transport proteins: glucose transporter 1 (GLUT1), CD36, and carnitine palmitoyltransferase Ia (CPT1a). These findings suggest that pioglitazone effectively reduces AF vulnerability by restoring atrial metabolism and mitigating mitochondrial damage [102]. Long-term treatment with pioglitazone has been shown to be both effective and generally well tolerated in patients with NASH who also have prediabetes or T2DM, offering sustained improvements in liver histology and metabolic parameters such as reduced hepatic triglyceride content, improvement in individual histological scores, including the fibrosis score, and upregulation of insulin sensitivity in adipose tissue, liver, and skeletal muscles [103]. However, pioglitazone is associated with several adverse effects, including hypoglycemia, weight gain, fluid retention, increased risk of skeletal fractures, potential hepatotoxicity, concerns about bladder cancer, and an elevated risk of atherosclerotic cardiovascular events or HF [37]. Table 3 presents summarized results from studies regarding thiazolidinediones effects in MASLD and AF.

#### 5.2.3. Statins

Statins are a class of drugs known as 3-hydroxy-3-methylglutaryl coenzyme A (HMG-CoA) reductase inhibitors, which work by blocking the enzyme responsible for cholesterol synthesis in the liver, effectively lowering blood cholesterol levels and reducing the risk of CVD [104]. Off-label applications of this group of medications include MASLD. As treatment with statins decreases also the hepatic production of apolipoprotein B-100 (apoB-100) containing lipoproteins, which results in downregulation of both cholesterol and triglyceride concentrations, they are successful in counteracting excessive accumulation of triglycerides in the liver—a feature characteristic for MASLD [37]. In a retrospective cohort study of 1238 patients with MASLD (mean age 53.7 ± 14.2 years; 63.8% men), disease severity was evaluated using the FIB-4 score. Over a follow-up period of 3.3 years on average, statin therapy was introduced in nearly half of the patients (47%), while 18% progressed to a high fibrosis risk category. Cox regression analysis revealed that statin exposure (estimated with statin prescription intensity) was significantly associated with a reduced risk of advancing to a high-risk FIB-4 category. Moderate exposure to statins showed a hazard ratio (HR) of 0.60 (95% CI: 0.42–0.84) and high exposure to statins revealed an HR of 0.61 (95% CI: 0.42–0.88). These associations remained robust even after adjusting for multiple confounders, including sex, race, marital status, smoking, BMI, HA, T2DM, cardiovascular disease, hypothyroidism, and CKD [105]. Complementing these findings, a larger cohort study, involving 7988 patients (mean age 53.0 ± 13.7 years; 41.8% men) across 16 tertiary referral centers, demonstrated that statin use correlated with a substantially lower long-term risk of all-cause mortality (aHR: 0.233; 95% CI: 0.127–0.426) as well as reduced progression of liver stiffness (HR: 0.542; 95% CI: 0.389–0.755) [106]. Statins, widely used for metabolic syndrome, have also demonstrated beneficial effects in AF [87]. A trial involving 105 patients, supported by a meta-analysis of 13 studies, demonstrated that atorvastatin is highly effective in reducing severity of oxidative stress, concentrations of inflammatory markers: serum high-sensitivity C-reactive protein (hs-CRP) and total cholesterol (TC) levels, as well as lowering the incidence of AF [107]. Table 4 presents summarized results from studies regarding statins effects in MASLD and AF.

#### 5.2.4. SGLT-2 Inhibitors

Sodium–glucose cotransporter 2 (SGLT-2) inhibitors are a class of antihyperglycemic agents that lower blood glucose by selectively blocking renal glucose reabsorption, thereby enhancing urinary glucose excretion [108]. Representatives of this class of medications, including dapagliflozin and empagliflozin, are well established for their cardiometabolic benefits. Emerging data hint at a possible inhibitory effect on the accumulation of hepatic fat content; however, this reduction appears limited, and its influence on progression of hepatic fibrosis remains an open question [109]. Alongside empagliflozin, dapagliflozin has been shown to improve hepatic steatosis and reduce serum levels of ALT and γ-glutamyltranspeptidase (GGT). Notably, its effects appear more pronounced in patients with advanced fibrosis, where it may also contribute to attenuation of such state, suggesting a potential dual benefit in both metabolic and structural liver parameters [110]. Furthermore, dapagliflozin has been associated with a significant promotion of weight loss and improved glycemic control, underscoring its multifaceted metabolic benefits [111]. A meta-analysis of 31 randomized controlled trials demonstrated a 25% relative risk reduction in serious AF events among patients treated with SGLT-2 inhibitors compared with placebo or other glucose-lowering agents [108]. In the Dapagliflozin Effect on Cardiovascular Events–Thrombolysis in Myocardial Infarction 58 (DECLARE-TIMI 58) trial, which enrolled 17,160 high-risk patients with T2DM, dapagliflozin reduced the incidence of reported AF and atrial flutter events by 19%. This effect was consistent irrespective of prior AF, atherosclerotic CVD, or HF status [112]. Findings regarding such outcomes were further supported by a meta-analysis of 39 randomized controlled trials including 107,770 participants, which also confirmed that SGLT-2 inhibitors significantly reduced the risk of AF and atrial flutter compared with placebo (RR 0.86; 95% CI, 0.77–0.95; I^2^ = 0%; *p* = 0.003). Moreover, it revealed that no significant difference was observed between groups receiving high- and low-dose SGLT-2 inhibitors (RR 0.78; 95% CI, 0.60–1.02; I^2^ = 0%; *p* = 0.07) [113]. This class of drugs can also ameliorate outcomes of invasive treatment measures of AF, as a prospective study conducted in China, supported by a meta-analysis, demonstrated that use of SGLT-2 inhibitors led to reduced risk of AF recurrence in patients with diabetes following ablation [114]. Although SGLT-2 inhibitors are known to counteract occurrence of AF, reduce hepatic lipid accumulation and may attenuate fibrosis through various metabolic and anti-inflammatory pathways, the use of dapagliflozin has also been associated with a risk of renal-related adverse events, warranting careful patient selection and monitoring [115]. Table 5 presents summarized results from studies regarding SGLT-2 inhibitors effects in MASLD and AF.

#### 5.2.5. Anticoagulants

Uncontrolled hepatic fat accumulation triggers a profibrotic response that promotes a pro-thrombotic state, often preceding clinically overt liver fibrosis [116]. Warfarin, a vitamin K antagonist (VKA), is widely used for the treatment and prevention of coagulopathic and thromboembolic disorders [117]. Direct oral anticoagulants (DOACs) are classified as direct thrombin inhibitors (dabigatran) or direct factor Xa inhibitors (rivaroxaban, apixaban, edoxaban, betrixaban). Compared with VKA, they offer rapid onset, predictable pharmacokinetics and pharmacodynamics, and fixed dosing without routine coagulation monitoring [118]. A retrospective study with a health research network (TriNetX) has shown that in patients with AF, coexisting NAFLD was linked to a more than twofold higher risk of both composite cardiovascular events (HR 2.17; 95% CI, 2.12–2.22) and hemorrhagic complications (HR 2.15; 95% CI, 2.05–2.56) [119]. The management of anticoagulated patients with liver disease is challenging due to both an elevated bleeding risk, driven by impaired hepatic synthetic function, variceal lesions, thrombocytopenia, and increased odds of ischemic events as well [120]. A randomized, double-blind trial—the Effective Anticoagulation with Factor Xa Next Generation in Atrial Fibrillation Thrombolysis in Myocardial Infarction Study 48 (ENGAGE AF-TIMI 48) trial, which enrolled 21,105 patients with AF, including 1083 participants (5.1%) with concomitant liver disease—found no significant difference in the risk of major bleeding or stroke between those treated with edoxaban and those receiving warfarin [121]. However, a meta-analysis of 3483 patients with AF and chronic liver disease demonstrated that DOAC therapy was associated with a significantly lower risk of major bleeding and a comparable risk of stroke relative to warfarin [122]. Another meta-analysis revealed that DOACs appeared to be associated with a relatively lower bleeding risk in patients with liver cirrhosis. It is worth noting that factors such as older age or class C of Child–Turcotte–Pugh scale of liver dysfunction negatively affected referenced phenomenon and increased risk of hemorrhage in DOAC-receiving subjects [123]. Table 6 presents summarized results from studies regarding anticoagulants effects in MASLD and AF.

#### 5.2.6. Resmetirom

The intricate interplay between dysregulated metabolism, chronic inflammation, and fibrogenesis in MASLD underscores the rationale for pharmacological interventions targeting multiple pathogenic pathways either through single agents with pleiotropic mechanisms of action or via combination therapies employing distinct compounds. The U.S. Food and Drug Administration (FDA) has recently granted approval for resmetirom, a thyroid hormone receptor β-selective agonist, marking it as the first pharmacological agent authorized for the treatment of MASH and associated liver fibrosis [124].

**Figure 4:**
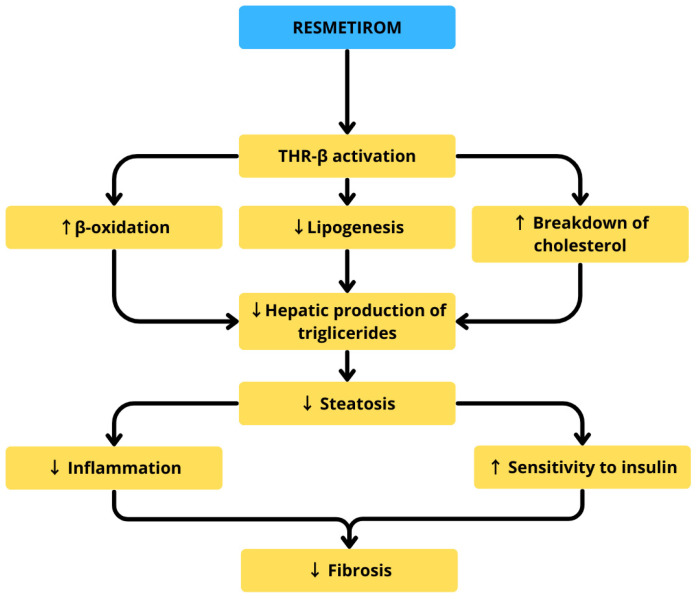
Resmetirom’s mechanism of action in treatment of MASH.

In a phase 3 trial (n = 966), 25.9–29.9% of patients treated with this medication achieved MASH resolution without worsening fibrosis, versus 9.7% in placebo [37]. Resmetirom has shown promising efficacy in reducing hepatic fat content and improving lipid parameters, while maintaining a favorable safety profile with generally mild side effects [36]. Thorough analyses of its pharmaceutical properties reveal that it exerts beneficial effects towards metabolic health in various mechanisms, including enhancement of hepatic mitochondrial β-oxidation, suppression of lipogenesis by affecting various intracellular signaling proteins and enzymes SREBP-1c, fatty acid synthase (FASN), and acetyl-CoA carboxylase 1 (ACC1), downregulation of low density lipoprotein (LDL), and reduction in fibrotic mediators such as TGF-β [36]. Figure 4 highlights resmetirom’s mechanism of action in treatment of MASH.

### 5.3. Steatosis Due to Amiodarone or Other Antiarrhythmic Drugs

**Figure 5:**
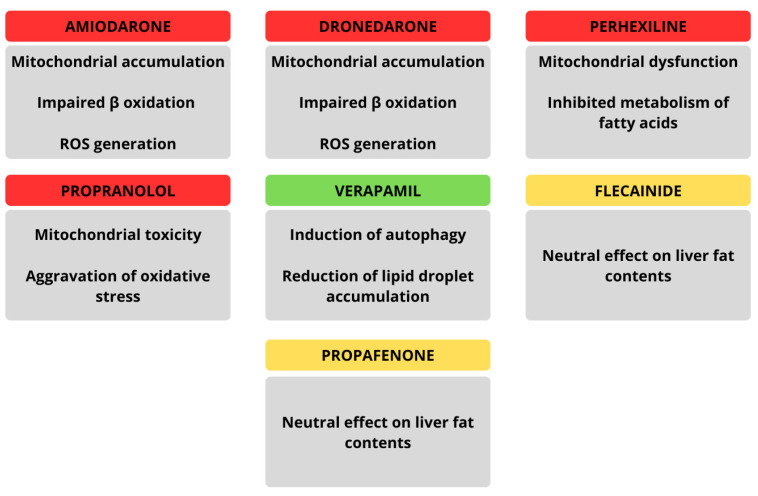
Summarized effects of particular antiarrhythmic drugs on hepatic tissue.

Amiodarone, a widely used antiarrhythmic drug and one of the possible therapeutic options for patients with AF, is one of the best-documented agents associated with drug-induced hepatic steatosis. Its cationic amphiphilic structure enables it to accumulate in mitochondria, where it disrupts β-oxidation of fatty acids and the electron transport chain, leading to both triglyceride accumulation and ROS generation. These mechanisms not only trigger macro- and micro-vesicular steatosis but can also progress to drug-induced steatohepatitis (DISH), fibrosis, and even cirrhosis with prolonged use [125]. Clinically, amiodarone-induced liver injury may manifest as asymptomatic transaminase elevations, jaundice, or, in rare cases, severe acute hepatitis. Other antiarrhythmic agents demonstrate similar steatogenic potential through mitochondrial toxicity. For example, dronedarone, a second-generation derivative, inhibits fatty acid β-oxidation but causes less electron transport disruption due to its shorter half-life, making severe mitochondrial injury less common. Perhexiline and propranolol have also been implicated in DISH via mechanisms involving mitochondrial dysfunction and oxidative stress [126]. Interestingly, some antiarrhythmic drugs such as verapamil may exert opposite effects: experimental models suggest verapamil can reduce lipid droplet accumulation and improve hepatic regeneration by enhancing autophagy, thus counteracting steatosis [127]. Collectively, while amiodarone and related compounds tend to promote hepatic fat accumulation through mitochondrial injury and impaired lipid handling, certain calcium channel blockers such as verapamil may have hepatoprotective, anti-steatotic effects. The main findings regarding effects exerted by particular antiarrhythmic drugs on hepatic tissue are highlighted in Figure 5.

## 6. Nutraceuticals’ Role in Prevention and Treatment of MASLD and AF

Nutraceuticals are bioactive compounds that provide health benefits beyond basic nutrition. Although they may originate from animals, microorganisms, or plants, a narrower definition limits this class to secondary metabolites obtained or enriched from plants [128,129]. Due to their structural diversity, nutraceuticals exert a wide range of biological activities that can beneficially modulate the course of numerous diseases. Compounds with antioxidant, anti-inflammatory, and metabolism-regulating properties are particularly promising for improving preventive and therapeutic strategies in MASLD and AF. Preclinical studies indicate that such compounds can inhibit disease progression and attenuate fibrosis in both the liver and heart [130,131,132]. However, the data from clinical trials are limited, with a vast majority of them regarding combined interventions with several nutraceuticals. Such an approach limits information on individual compounds’ effects for MASLD or AF. Moreover, the fact that most nutraceuticals exist in various, closely related forms or were administered in different dosage regimens makes comparing particular trials more complex and further limits their significance. It should be noted that none of the compounds discussed below are approved for general use in guideline-directed cardiometabolic therapy [132,133].

### 6.1. Omega-3 Polyunsaturated Fatty Acids

Omega-3 polyunsaturated fatty acids (Ω-3 PUFAs), primarily derived from fish and seafood, exert favorable effects on lipid metabolism by modulating PPARs, sterol regulatory element-binding protein-1c (SREBP-1c), and carbohydrate response element-binding protein (ChREBP) activity, thereby alleviating dyslipidemia. In MASLD patients, 12 months of eicosapentaenoic acid (EPA) and docosahexaenoic acid (DHA) supplementation (3.6 g/day) significantly decreased gamma-glutamyl transferase (GGT) levels [134,135]. Nogueira et al. showed that combination of EPA and DHA with alpha-linolenic acid (ALA) was capable of reducing triglycerides levels in MASH-affected patients. Moreover, individuals who displayed elevations in specific Ω-3 PUFAs in their blood plasma presented also mitigation of liver lobular inflammation, steatosis, and ballooning—histological exponents of progressive disease process in MASLD and MASH [136]. Despite strong cardioprotective and anti-inflammatory properties, several clinical trials using 1–4 g/day of Ω-3 PUFAs found no significant effects on inflammatory cytokines (IL-6, IL-8, IL-10, TNF-α, MCP-1) or recurrence rates of paroxysmal or persistent AF [137,138]. Similar null results were reported in subjects suffering from heart failure [139]. However, perioperative Ω-3 PUFA administration significantly reduced postoperative AF incidence in patients undergoing coronary artery bypass graft (CABG), including those with a prior history of myocardial infarction [140,141].

### 6.2. Vitamin E

The term vitamin E refers to a group of tocochromanols: α-, β-, γ- and δ-tocopherols with their corresponding tocotrienols. Vitamin E’s beneficial role for human health is predominantly associated with its potential to act as a lipophilic radical-scavenging antioxidant [142,143]. In non-diabetic patients suffering from MASH, a 96-week long administration of vitamin E at dose of 300 mg/day was shown to positively affect liver histological features, alleviating lobular inflammation, steatosis, and fibrosis score. Subjects treated with this antioxidant agent also displayed significantly decreased ALT and AST levels in blood plasma as well as diminished activity of IL-6 [144]. Similar results were acquired by Al-Baiaty et al. in an examination of tocotrienol-rich fraction vitamin E in a pediatric population. Supplementation with 50 mg of this nutraceutical, for 6 months, led to reductions in apolipoprotein A1, AST, and parameters resembling damage of DNA among children participating in the trial. Moreover, ultrasonographic assessment revealed alleviation of hepatic steatosis [145]. As in case of other nutraceuticals referenced in this review, the data regarding their role in preventing development of AF are limited to findings from patients undergoing cardiac surgeries and concern postoperative AF. Moreover, available studies were conducted using combination of vitamin E and other antioxidant agents or nutraceuticals, which makes it impossible to determine how much vitamin E contributed to the trials’ outcomes. Combined preparations of Ω-3 PUFAs, vitamin C, and vitamin E showed efficacy in preventing incidence of postoperative AF and improving the activity of antioxidant enzymes (CAT, SOD, and GPX) [146]. This beneficial potential was shown to be more pronounced among elderly populations, especially subjects older than 60 years [147].

### 6.3. Polyphenols

Polyphenols, a diverse class of plant-derived secondary metabolites, have garnered attention for their broad antioxidant and anti-inflammatory potential in disease prevention and therapy. In MASLD patients with coexisting T2DM, curcumin (1500 mg/day) significantly improved antioxidant enzyme activity (GPX, SOD) and reduced inflammatory cytokines (IL-1, IL-6, TNF-α), resulting in decreased body fat, BMI, hepatic steatosis, and liver stiffness [148]. However, in a trial involving CABG patients, curcumin combined with piperine did not prevent AF, although it reduced CRP levels and enhanced total antioxidant capacity [149]. In contrast, quercetin (1000 mg/day) administered two days preoperatively and for seven days post-CABG significantly lowered AF incidence. The effect was attributed to improved endothelial sensitivity to acetylcholine and attenuation of cellular senescence and inflammaging pathways [150]. Among MASLD patients, quercetin (500 mg/day for 12 weeks) modestly reduced body weight, fat mass, BMI, and intrahepatic lipid content (by approximately 2%), with the strongest effect observed in individuals with lower baseline hepatic lipid levels [151]. Although resveratrol is a well-established polyphenol with general health benefits, clinical trials in MASLD populations have shown its capability to induce only modest improvements in body weight, waist circumference, and liver fat, without significant changes in serum lipid profiles, liver enzymes, or cardiovascular risk markers, including atherogenic indices and blood pressure [152,153,154].

## 7. Conclusions

The growing prevalence of MASLD and AF underscores the urgent need to recognize their close interconnection. Both conditions share a constellation of cardiometabolic risk factors—including obesity, T2DM, dyslipidemia, and HA—and are increasingly understood as two manifestations of systemic metabolic dysfunction. Current evidence highlights that MASLD not only predisposes individuals to the development of AF but also worsens its recurrence and complications, while AF itself may accelerate hepatic injury through chronic inflammation, oxidative stress, and impaired hemodynamics.

The clinical significance of this bidirectional relationship is profound. Individuals with coexisting MASLD and AF face increased risks of unfavorable cardiovascular events, including stroke and HF. They are also at greater risk of liver fibrosis progression and present higher overall mortality. Importantly, this calls for an integrated diagnostic and therapeutic strategy. The use of non-invasive biomarkers and imaging techniques facilitates early detection of advanced fibrosis and arrhythmic risk, while structured risk stratification should guide multidisciplinary care. Lifestyle interventions, particularly sustained weight loss and metabolic optimization, remain the cornerstone of management with proven benefits in both liver and cardiac outcomes.

Pharmacological strategies such as utilization of GLP-1 receptor agonists, SGLT-2 inhibitors, statins, and thiazolidinediones have shown promise in simultaneously addressing hepatic steatosis and reducing AF burden. Furthermore, appropriate anticoagulation and rhythm-control strategies must be carefully tailored to MASLD patients to balance thromboembolic risk with hepatic safety. Nevertheless, most current evidence is derived from observational studies, and large-scale randomized trials are required to establish causality and refine therapeutic recommendations.

Future research should focus on clarifying the molecular mediators of the liver–heart axis, validating novel biomarkers for risk prediction, and developing multidisciplinary treatment models that bridge hepatology, cardiology, endocrinology, and primary care. Ultimately, recognizing MASLD as a systemic disease with significant cardiovascular consequences represents a critical step toward improving outcomes, reducing mortality, and lessening the global healthcare burden associated with both MASLD and AF.

## Figures and Tables

**Table 1:** Comparison of methodological, populational and outcome differences in studies assessing coexistence of MASLD and AF.

First Author, Year	Population and AF Outcome (Incident/Prevalent/Recurrence)	Exposure Definition (NAFLD/MASLD; Enzymes vs. Imaging vs. Histology)	Adjustments	Effect Size (95% CI)	Heterogeneity (I^2^)/Publication Bias	Stated Limitations/Notes
Kang et al., 2020 [17]	Health-screen cohort *n* = 6293 with US-defined NAFLD; prevalent AF (ECG)	Ultrasound; advanced fibrosis by NFS/FIB-4	Sex, HA, obesity; + glucose, GGT, hs-CRP; + lipids	Advanced fibrosis (high cut-offs): OR 3.84–12.29 for prevalent AF	NA	Cross-sectional; single ECG may miss paroxysmal AF
Pastori et al., 2020 [23]	AF clinic cohort n = 1735 with OAC; prevalent AF (cross-sectional) + incident CV outcomes	NAFLD by FLI ≥ 60	Age, sex, BMI, HA, T2DM, HF, smoking, prior CV/CVA, renal function, meds	Bleeding HR 0.85 (0.52–1.37); CV events HR 0.91 (0.64–1.29)	NA	FLI surrogate; White European; not AF incidence study
Riley et al., 2024 [24]	TriNetX EHR > 114 M; incident AF among CV outcomes; MASLD ± T2DM	ICD-based MASLD with metabolic criteria	Propensity matching incl. age, sex, BMI, HA, IHD, HF, CKD, cirrhosis, meds; HbA1c in diabetes	T2D + MASLD vs. MASLD: AF HR 1.09 (1.03–1.16); MASLD in T2D: AF HR 0.97 (0.85–1.11)	NA	ICD-based misclassification; unmeasured confounding; medication time-updating absent
Simon et al., 2023 [25]	Nationwide matched cohort (Sweden) *n* = 11,206 biopsy-confirmed MASLD + 51,856 controls; incident AF	Histology (steatosis→cirrhosis)	Age, sex, education, T2DM, obesity, HA, lipids, CKD, hospitalizations, alcohol disorder + meds (sens.)	aHR AF 1.26 (1.18–1.35); rises to 1.59 (1.15–2.19) in cirrhosis	NA (single study); sibling/sensitivity analyses robust	Residual confounding; limited ethnic diversity; possible undiagnosed MASLD in controls
Brunner et al., 2019 [26]	Narrative systematic review; no direct AF endpoint	Imaging/biopsy/biomarkers across cited studies	Varied; typically, age, sex, BMI, T2DM, HA, lipids, alcohol	Weight loss ≥ 10% linked with NASH resolution; fibrosis regression ~45% (not AF-specific)	NA	No pooled estimates; heterogeneous designs; AF not specifically assessed
Cho et al., 2025 [28]	Nationwide T2DM cohort *n* ≈ 2.48 M; incident AF	FLI ≥ 60 + metabolic risk to define MASLD; categories incl. MetALD/ALD	Age, sex, income, lifestyle, BMI, DM duration/therapy, CKD, ASCVD, CHA_2_DS_2_-VASc	MASLD aHR 1.10 (1.08–1.11); MetALD 1.26 (1.22–1.29); ALD + met 1.48 (1.41–1.55)	NA (single cohort)	FLI-based; possible AF underdiagnosis; Asian-only cohort
Targher et al., 2011 [29]	Narrative review (diabetics); CVD and CKD (AF not isolated)	Imaging/enzymes; 1 biopsy study in cited works	Age, sex, BMI/waist, BP, HbA1c, lipids, meds; sometimes HOMA-IR	Representative: incident CVD HR ~1.87; CKD HR ~1.49 (study-level)	NA	Heterogeneous definitions; observational; AF not specifically studied
Jaiswal et al., 2023 [30]	Meta-analysis of 12 cohorts (~18 M); incident AF	NAFLD by imaging (US/CT) or ICD codes	Age, sex, BMI/obesity, HA, T2DM, lipids, smoking	RR 1.42 (1.191.68) for AF; stroke 1.26 (1.16–1.36); HF 1.43 (1.03–2.00)	AF I^2^ = 91% (high); funnel plots~symmetric	Variable NAFLD definitions; residual confounding; limited fibrosis data
Mantovani et al., 2024 [31]	Meta-analysis of 16 cohort studies; outcome: incident AF	NAFLD/MASLD by imaging, FLI/HSI, ICD codes; 1 study biopsy	Most studies adjusted for age, sex, BMI, T2DM, HA, lipids, smoking/alcohol	Pooled HR 1.18 (1.10–1.32) overall; fully adjusted ≈ 1.11 (1.05–1.18)	I^2^ ≈ 92% (primary), subgroups lower; Egger *p* > 0.05 (no bias)	Observational data; heterogeneity; limited histology-based evidence
Wang et al., 2025 [32]	Single-center prospective ablation cohort *n* = 303; outcome: AF recurrence post-cryoballoon (median 14 mo)	MASLD: FLI ≥ 60 + ≥1 metabolic risk factor	Age, sex, AF type/duration, LAD, LVEF, alcohol, CHD, HF	AF recurrence: HR 2.24 (1.35–3.74); paroxysmal 2.38; persistent 2.55	NA (single-center)	No imaging/biopsy; short follow-up; residual confounding
Moon et al., 2023 [33]	Nationwide cohort *n* = 351,068; primary CVD composite (AF not primary)	Steatosis by FLI; MASLD/MetALD/ALD per 2023 consensus	Age, sex, BMI, income, HA, T2DM, lipids, smoking, alcohol, activity, meds; competing risks	Incident CVD: MASLD SHR 1.19 (1.15–1.24); MetALD 1.28; ALD 1.29	NA	FLI-based diagnosis; Asian-only; AF not isolated as endpoint

Abbreviations: AF, atrial fibrillation; aHR, adjusted hazard ratio; ALD, alcohol-related liver disease; ASCVD, atherosclerotic cardiovascular disease; BMI, body mass index; CHA_2_DS_2_-VASc, congestive heart failure, hypertension, age ≥ 75, diabetes, stroke, vascular disease, age 65–74, sex; CHD, coronary heart disease; CI, confidence interval; CKD, chronic kidney disease; CT, computed tomography; CV, cardiovascular; CVA, cerebrovascular accident; ECG, electrocardiography; EHR, electronic health record; FLI, fatty liver index; GGT, gamma-glutamyl transferase; HA, hypertension; HF, heart failure; HR, hazard ratio; hs-CRP, high-sensitivity C-reactive protein; HSI, hepatic steatosis index; I^2^, I-squared statistic; ICD, international classification of diseases; IHD, ischemic heart disease; LAD, left atrial diameter; LVEF, left ventricular ejection fraction; MASLD, metabolic dysfunction-associated steatotic liver disease; MetALD, metabolic dysfunction-associated alcohol-related liver disease; NA, not available; NAFLD, non-alcoholic fatty liver disease; NASH, non-alcoholic steatohepatitis; RR, relative risk; SHR, sub-hazard ratio; T2DM, type 2 diabetes mellitus; US, ultrasound.

**Table 2:** Summarized results of studies regarding GLP-1 receptor agonists effects in MASLD and AF.

Type of Study	Examined Medication(s)/Intervention(s)	Participants	Results	Ref.
Phase 2 clinical trial (LEAN)	Liraglutide (1.8 mg/day administered by subcutaneous injection)	52 overweight participants with NASH monitored for 48 weeks	improved NASH resolution (↓ steatosis, ↓ hepatocyte ballooning) decreased risk of fibrosis progression decreased levels of ALT and GGT in blood plasma decreased body weight and BMI values improved HbA1c values	[93]
Systematic review and meta-analysis of RCTs	Semaglutide (oral 14 mg/day; subcutaneous 0.5–1.0 mg weekly, up to 2.4 mg weekly in some trials)	10 randomized clinical trials; 12,651 total participants (7285 on semaglutide, 5366 on placebo); median follow-up ~68 months	decreased incidence of AF by 42% in individuals with high cardiovascular risk	[95]
Systematic review and meta-analysis (propensity-matched observational studies)	GLP-1 receptor agonists (liraglutide 1.2–1.8 mg daily; semaglutide 0.5–1.0 mg weekly; dulaglutide 0.75–1.5 mg weekly)	3 studies; 6031 participants after AF ablation; follow-up: 12 months	decreased risk of AF recurrence after ablation (HR = 0.55)	[96]
Real-world cohort study	GLP-1 receptor agonists (liraglutide 1.2–1.8 mg daily; semaglutide 0.5–1.0 mg weekly; dulaglutide 0.75–1.5 mg weekly) vs. DPP-4i vs. SGLT-2 inhibitors	14,566 participants GLP-1RA vs. DPP4i; 9424 participants GLP-1RA vs. SGLT-2 inhibitors; follow-up: 3–4 years	decreased risk of new AF in GLP-1RA group vs. DPP4i group (HR = 0.82).	[97]

Abbreviations: AF, atrial fibrillation; ALT, alanine aminotransferase; BMI, body mass index; DPP4i, dipeptidyl peptidase 4 inhibitors; GGT, gamma-glutamyl transferase; GLP-1, glucagon-like peptide 1; GLP-1RA, glucagon-like peptide 1 receptor agonist; HbA1c, hemoglobin A1c; HR, hazard ratio; LEAN, Liraglutide safety and efficacy in patients with non-alcoholic steatohepatitis; NASH, non-alcoholic steatohepatitis; RCTs, randomized controlled trials; SGLT-2, sodium–glucose cotransporter 2.

**Table 3:** Summarized results of studies regarding thiazolidinediones effect in MASLD and AF.

Type of Study	Examined Medication(s)/Intervention(s)	Participants/Animal Model	Results	Ref.
Randomized, placebo-controlled proof-of-concept trial	Pioglitazone (45 mg daily, administered orally) + hypocaloric diet	55 patients with impaired glucose tolerance or T2DM and NASH monitored for 6 months	improved resolution of steatosis, ballooning necrosis, and inflammation in liver decreased blood plasma levels of AST and ALT decreased hepatic fat content beneficial effects were stronger in patients receiving pioglitazone + hypocaloric diet than in group receiving sole pioglitazone statistically significant	[98]
Meta-analysis of RCTs and observational studies	Thiazolidinediones, especially pioglitazone (15–45 mg/day administered orally) but also rosiglitazone (4–8 mg/day administered orally)	7 studies (3 RCTs + 4 observational); 130,854 diabetic participants (11,781 thiazolidinediones users, 119,073 controls); follow-up ranging between 30 days and 12 years	decreased risk of incident AF among thiazolidinediones users (OR = 0.73) decreased risk of new-onset AF among thiazolidinediones users: (OR = 0.77) decreased risk of recurrent AF among thiazolidinediones users: (OR = 0.41)	[100]
Observational study	pioglitazone (30 mg/day administered orally)	101 participants with PAF and T2DM undergoing catheter ablation monitored for 15 months	improved preservation of sinus rhythm decreased AF recurrence decreased rate of re-ablation	[101]
Preclinical in vivo (animal) study	pioglitazone (administered orally for 2 weeks)	rats immunized with β1-adrenergic receptor peptide to induce AF susceptibility	decreased AF vulnerability improved atrial structure and metabolism	[102]

Abbreviations: AF, atrial fibrillation; ALT, alanine aminotransferase; AST, aspartate aminotransferase; NASH, non-alcoholic steatohepatitis; OR, odds ratio; PAF, paroxysmal atrial fibrillation; RCTs, randomized controlled trials; T2DM, type 2 diabetes mellitus.

**Table 4:** Summarized results from studies regarding statins effects in MASLD and AF.

Type of Study	Examined Medication(s)/Intervention(s)	Participants	Results	Ref.
Retrospective cohort study	Statins (exposure estimated based on medical prescriptions, exact preparations names and doses not specified)	1238 patients with MASLD, with baseline FIB-4 < 2.67 (determining low or indeterminate fibrosis risk); mean follow-up: 3.3 years	moderate and high intensity of statin prescription decreased risk of progression to high-risk FIB-4 (HR = 0.6 and 0.61, respectively)	[105]
Longitudinal, multi-center cohort study	Statins (simvastatin, atorvastatin, pravastatin, rosuvastatin, fluvastatin, lovastatin, pitavastatin; exact dosage not specified)	7988 patients with MASLD having ≥ 2 VCTE (baseline LSM median 5.9 kPa IQR 4.6–8.2); mean follow-up: 4.6 years	statin usage reduced all-cause mortality (aHR = 0.233) statin usage reduced liver-related events (aHR = 0.380) statin usage reduced liver stiffness progression (cACLD: HR = 0.542; non-cACLD: HR = 0.450)	[106]
Prospective, controlled clinical trial	Atorvastatin (20 mg/day administered orally)	105 participants with AF receiving amiodarone, monitored for 12 months	decreased levels of hs-CRP and TC in blood plasma decreased recurrence of AF	[107]

Abbreviations: AF, atrial fibrillation; aHR, adjusted hazard ratio; cACLD, compensated advanced liver disease; FIB-4, fibrosis-4 index; HR, hazard ratio; hs-CRP, high-sensitivity C-reactive protein; IQR, interquartile range; MASLD, metabolic dysfunction-associated steatotic liver disease; TC, total cholesterol; VCTE, vibration-controlled transient elastography.

**Table 5:** Summarized results from studies regarding SGLT-2 inhibitors effects in MASLD and AF.

Type of Study	Examined Medication(s)/Intervention(s)	Participants	Results	Ref.
Observational study	SGLT-2 inhibitors (59.2% dapagliflozin, 40.0% empagliflozin, 0.8% canagliflozin, <0.1% ertugliflozin; exact dosage not specified)	136,956 participants with T2DM (79,343 new users of SGLT-2 inhibitors vs. 57,613 new users of GLP-1 receptor agonists); follow-up: 0.7–3.8 years	Decreased risk of new-onset AF in participants using SGLT-2 inhibitors when compared with participants using GLP-1 receptor agonists (aHR = 0.89)	[108]
Randomized, active-controlled, open label trial	dapagliflozin (5 mg/day administered orally)	57 participants with T2DM and NAFLD monitored for 24 weeks	decreased steatosis in liver decreased visceral fat accumulation decreased liver stiffness in group with significant liver fibrosis (baseline LSM ≥ 8.0 kPa)	[110]
Sub-analysis of DECLARE-TIMI 58 RCT	dapagliflozin (10 mg/day administered orally)	17,160 participants with T2DM and high risk of CVD; median follow-up: 4.2 years	decreased incidence of AF/AFL adverse events	[112]
Systematic review and meta-analysis, RCTs ≥ 52 weeks follow-up	SGLT-2 inhibitors (dapagliflozin 5–10mg/day; empagliflozin 10–25 mg/day; canagliflozin 100–300 mg/day; ertugliflozin 5–15 mg/day; sotagliflozin 200–400 mg/day; administered orally)	39 RCT studies (107,770 users of SGLT-2 inhibitors); follow-up: ≥52 weeks	decreased risk of AF/AFL among SGLT-2 users (RR = 0.86) no statistically significant difference between high- vs. low-dose groups, though trend favoring high dose. no significant impact on VT, VF, or sinus bradycardia	[113]
Propensity-score matched observational study + meta-analysis	SGLT-2 inhibitors (exact preparation and dosage not specified)	525 participants with T2DM after AF catheter ablation; follow-up: 18 months	decreased AF recurrence among SGLT-2 users (HR = 0.63)	[114]
Meta-analysis of prospective observational studies	SGLT-2 inhibitors (canagliflozin, dapagliflozin, tofogliflozin or empagliflozin; dosage not specified)	4715 participants with T2DM after AF catheter ablation	decreased AF recurrence among SGLT-2 users (HR = 0.61)	[115]

Abbreviations: AF, atrial fibrillation; AFL, atrial flutter; aHR, adjusted hazard ratio; CVD, cardiovascular disease; DECLARE-TIMI 58 RCT, Dapagliflozin Effect on Cardiovascular Events–Thrombolysis in Myocardial Infarction 58; GLP-1, glucagon-like peptide 1; HR, hazard ratio; LSM, liver stiffness measurement; NAFLD, non-alcoholic fatty liver disease; RCTs, randomized controlled trials; RR, relative risk; SGLT-2, sodium–glucose cotransporter 2; T2DM, type 2 diabetes mellitus; VF, ventricular fibrillation; VT, ventricular tachycardia.

**Table 6:** Summarized results from studies regarding anticoagulants effects in MASLD and AF.

Type of Study	Examined Medication(s)/Intervention(s)	Participants	Results	Ref.
Sub-analysis of the ENGAGE AF-TIMI 48 RCT	Edoxaban (15–60 mg/day; administered orally) vs. warfarin (doses adjusted to INR of 2–3; administered orally)	21,105 participants with AF (1083 with history of liver disease); mean follow-up: 2.8 years	Among AF patients, those with liver disease had similar efficacy of edoxaban vs. warfarin for preventing systemic embolic events, but had increased bleeding risk compared with patients without liver disease (aHR for major bleeding = 1.38 for those with liver disease vs. no liver disease)	[121]
Systematic review and meta-analysis of observational/cohort studies	DOACs, warfarin, or LMWH (exact dosage not specified)	43,532 participants with advanced liver disease or cirrhosis (27,574 on DOACs; 15,958 on warfarin or LMWH); follow-up: mean or median time not specified	Decreased risk of major bleeding (HR = 0.39) and intracranial hemorrhage (HR = 0.48) among DOAC users when compared with warfarin/LMWH users.	[122]

Abbreviations: AF, atrial fibrillation; aHR, adjusted hazard ratio; DOACs, direct oral anticoagulants; ENGAGE AF-TIMI 48, Effective Anticoagulation with Factor Xa Next Generation in Atrial Fibrillation–Thrombolysis in Myocardial Infarction Study 48; HR, hazard ratio; INR, international normalized ratio; LMWH, low-molecular-weight heparin; RCT, randomized controlled trial.

## Data Availability

No new data were created or analyzed in this study.
